# First report of the use of a digital single-operator cholangioscope for endoscopic direct diverticulitis therapy

**DOI:** 10.1055/a-2316-1111

**Published:** 2024-06-06

**Authors:** Jun Cai, Silin Huang, Yangbor Lu, Suhuan Liao, Guang Yang, Bo Li, Jianzhen Ren

**Affiliations:** 1 Gastroenterology, South China Hospital, Medical School, Shenzhen University, Shenzhen, China; 2 Gastroenterology and Hepatology, Xiamen Chang Gung Memorial Hospital, Huaqiao University, Xiamen, China

A 31-year-old man with a 2-day history of periumbilical abdominal pain was evaluated at our hospital. Abdominal computed tomography (CT) revealed ileocecal inflammation and multiple cecal diverticula ( Fig. 1 **a** ). Colonoscopy identified several diverticula in the cecum, particularly one manifesting inflamed and edematous mucosa coated in yellowish-white purulent exudate ( Fig. 1 **b** ). A continuous purulent discharge was noted.

**Fig. 1:**
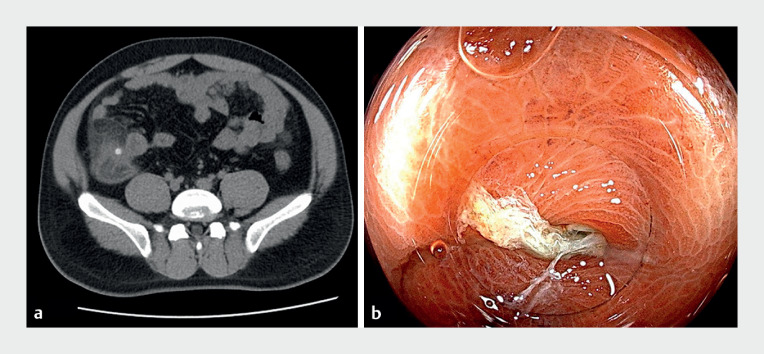
**a** Computed tomography (CT) revealed ileocecal inflammation, multiple cecal diverticula, and fecaliths. **b** Colonoscopy identified the diverticula in the cecum especially one with inflamed and edematous mucosa coated in yellowish-white purulent exudate.

Upon obtaining informed consent, we embarked upon endoscopic direct diverticulitis therapy (EDDT), using a digital single-operator cholangioscope (DSOC) (EyeMax, 3.3 mm; Micro-Tech, Nanjing, China). This revealed a substantial volume of fecaliths in the diverticular cavity ( Fig. 2 **a** , Video 1 ). These fecaliths were meticulously fragmented, extracted, and removed using a disposable basket ( Fig. 2 **b** ) and biopsy forceps ( Fig. 2 **c** ), following repeated lavages with metronidazole and sodium chloride. This left the diverticular mucosa cleansed though characterized by roughness and swelling, without evidence of perforation ( Fig. 2 **d** ). A 7-Fr pancreatic duct stent was strategically placed to ensure unobstructed drainage ( Fig. 3 ).

**Fig. 2:**
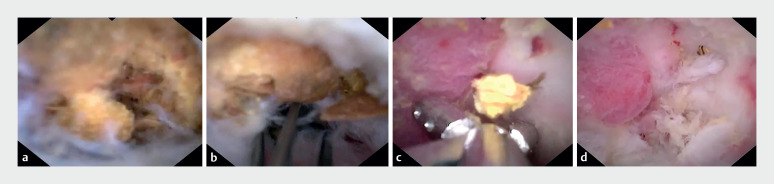
Endoscopic treatment of diverticulitis using a digital single-operator cholangioscope (DSOC). **a** A considerable volume of fecaliths could be seen within the cecal diverticular cavity. **b** The fecaliths were carefully fragmented, extracted, and removed using a disposable basket. **c** Several fecaliths were extracted using a biopsy forceps. **d** The cleared diverticular cavity.

Initial report on the use of a digital single-operator cholangioscope for endoscopic direct diverticulitis therapy (EDDT).Video 1

**Fig. 3:**
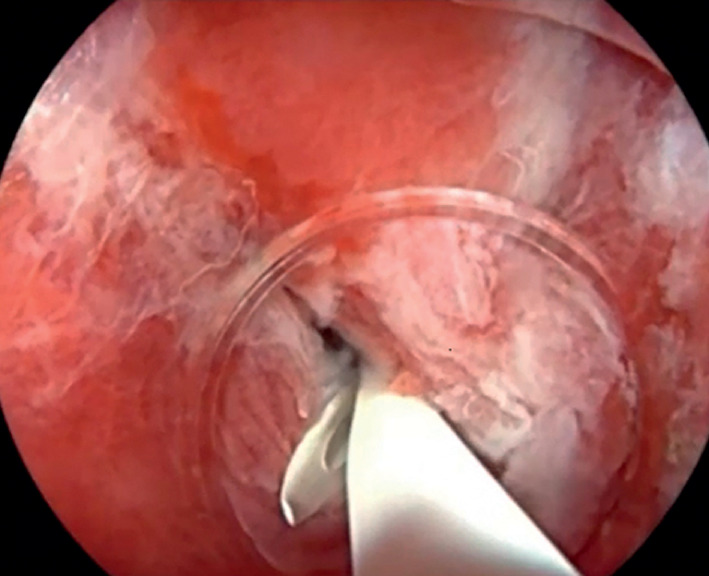
A stent was strategically placed to ensure unobstructed drainage.

The patient’s postoperative course was marked by rapid alleviation of abdominal discomfort, a decline in routine hematological and C-reactive protein (CRP) levels, and diminished inflammation on subsequent CT imaging ( Fig. 4 ).

**Fig. 4:**
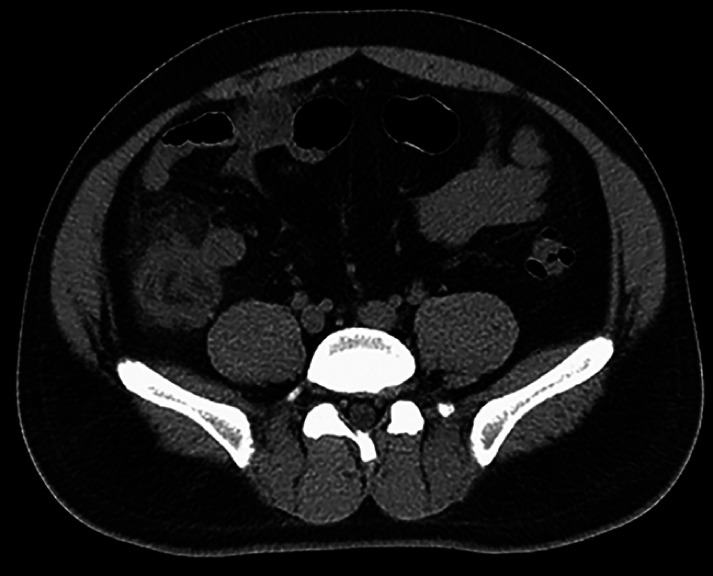
Postoperative CT demonstrated the reduction in the severity of ileocecal inflammatory exudation compared to before the procedure.

Acute diverticulitis is mainly treated through medication and surgical intervention 1 . The use of a DSOC has proven effective for managing inflammations in natural conduits such as the bile duct, pancreatic duct, and appendix 2 3 . To the best of our knowledge, this is the first report of the use of a DSOC to treat acute diverticulitis, illustrating its potential for precise, minimally invasive therapy. The direct visualization and management of diverticular contents offers a safe, efficient alternative to traditional interventions, with the promise of shorter hospital stays and rapid recovery. This novel EDDT approach could reshape the management of acute diverticulitis, emphasizing the importance of technological integration into endoscopic practice.

Endoscopy_UCTN_Code_TTT_1AQ_2AF

## Notes

Endoscopy E-Videos https://eref.thieme.de/e-videos
*E-Videos* is an open access online section of the journal *Endoscopy* , reporting on interesting cases and new techniques in gastroenterological endoscopy. All papers include a high-quality video and are published with a Creative Commons CC-BY license. Endoscopy E-Videos qualify for HINARI discounts and waivers and eligibility is automatically checked during the submission process. We grant 100% waivers to articles whose corresponding authors are based in Group A countries and 50% waivers to those who are based in Group B countries as classified by Research4Life (see: https://www.research4life.org/access/eligibility/ ). This section has its own submission website at https://mc.manuscriptcentral.com/e-videos .
